# A novel loop-mediated isothermal amplification-lateral-flow-dipstick (LAMP-LFD) device for rapid detection of *Toxoplasma gondii* in the blood of stray cats and dogs

**DOI:** 10.1051/parasite/2021039

**Published:** 2021-05-03

**Authors:** Yangji Xue, Qingming Kong, Haojie Ding, Chengzuo Xie, Bin Zheng, Xunhui Zhuo, Jianzu Ding, Qunbo Tong, Di Lou, Shaohong Lu, Hangjun Lv

**Affiliations:** 1 Department of Immunity and Biochemistry, Institute of Parasitic Disease, Zhejiang Academy of Medical Sciences 310013 Hangzhou PR China; 2 Hangzhou Medical College 310053 Hangzhou PR China

**Keywords:** *Toxoplasma gondii*, Diagnosis, 529-RE, LAMP, LFD, Prevalence

## Abstract

*Toxoplasma gondii* is an obligate intracellular protozoan parasite that causes toxoplasmosis and threatens warm-blooded animal and human health worldwide. Simple and applicable diagnostic methods are urgently needed to guide development of effective approaches for prevention of toxoplasmosis. Most molecular diagnostic tools for *T. gondii* infection require high technical skills, sophisticated equipment, and a controlled lab environment. In this study, we developed a loop-mediated isothermal amplification-lateral-flow-dipstick (LAMP-LFD) assay that specifically targets the 529 bp for detecting *T. gondii* infection. This novel portable device is universal, fast, user-friendly, and guarantees experimental sensitivity as well as low risk of aerosol contamination. Our LAMP-LFD assay has a detection limit of 1 fg of *T. gondii* DNA, and shows no cross-reaction with other parasitic pathogens, including *Cryptosporidium parvum*, *Leishmania donovani*, and *Plasmodium vivax*. We validated the developed assay by detecting *T. gondii* in DNA extracted from blood samples collected from 318 stray cats and dogs sampled from Deqing, Wenzhou, Yiwu, Lishui and Zhoushan cities across Zhejiang province, Eastern China. The LAMP-LFD device detected *T. gondii* DNA in 4.76 and 4.69% of stray cats and dogs, respectively. In conclusion, the developed LAMP-LFD assay is efficient, minimizes aerosol contamination, and is therefore suitable for detecting *T. gondii* across basic medical institutions and field settings.

## Abstract (fr)

*Toxoplasma gondii* est un parasite protozoaire intracellulaire obligatoire qui provoque la toxoplasmose et menace la santé humaine et les animaux à sang chaud dans le monde entier. Des méthodes de diagnostic simples et applicables sont nécessaires de toute urgence pour guider le développement d’approches efficaces pour la prévention de la toxoplasmose. La plupart des outils de diagnostic moléculaire pour l’infection par *T. gondii* nécessitent des compétences techniques élevées, un équipement sophistiqué et un environnement de laboratoire contrôlé. Dans cette étude, nous avons développé un test par bandelettes à flux latéral d’amplification isotherme médiée par les boucles (LAMP-LFD) qui cible spécifiquement les 529 pb qui détectent une infection par *T. gondii*. Ce nouvel appareil portable est universel, rapide, convivial et garantit une sensibilité expérimentale ainsi qu’un faible risque de contamination par aérosol. Notre test LAMP-LFD a une limite de détection de 1 fg d’ADN de *T. gondii* et ne montre aucune réaction croisée avec d’autres pathogènes parasites, y compris *Cryptosporidium parvum*, *Leishmania donovani* et *Plasmodium vivax*. Nous avons validé le test en détectant *T. gondii* dans l’ADN extrait d’échantillons de sang prélevés sur 318 chats et chiens errants prélevés dans les villes de Deqing, Wenzhou, Yiwu, Lishui et Zhoushan dans la province du Zhejiang, dans l’est de la Chine. Le dispositif LAMP-LFD a détecté la prévalence de l’ADN de *T. gondii* chez respectivement 4,76 et 4,69% des chats et chiens errants. En conclusion, le test LAMP-LFD développé est efficace, minimise la contamination par les aérosols et convient donc à la détection de *T. gondii* dans les établissements médicaux simples et sur le terrain.

## Introduction

*Toxoplasma gondii* is an obligate intracellular protozoan parasite that infects a wide variety of warm-blooded animals, including humans, causing toxoplasmosis [3]. *Toxoplasma gondii* infections are distributed worldwide, with an estimated one-third of the global population reportedly seropositive, although the infection rates vary significantly by geographical region [11]. A recent analysis showed reported antibody positive rates of 8.20 and 8.60% for *T. gondii* in the general population and pregnant women across China, respectively [6, 19]. *Toxoplasma gondii* infection in immunocompetent individuals is usually subclinical, with symptoms such as malaise, fever, myalgias, and isolated cervical or occipital lymphadenopathy. Furthermore, pregnant women infected with *T. gondii* during pregnancy are predisposed to miscarriages, stillbirths, and fetal abnormalities, and newborns may present ocular and neurological lesions [2]. According to two meta-analyses, the seroprevalence of *T. gondii* among cats and dogs in mainland China was 20.3% and 11.1%, respectively [5, 9]. Meanwhile, the seropositivity rate in stray cats is higher than in pet cats [5]. Cats as definitive hosts or final hosts of *T. gondii* can excrete infective *T. gondii* oocysts in their feces, thus threatening the environment, humans and other animals. Also, there is no effective medication or vaccine for *T. gondii* infection in cats or dogs. Therefore, effective, rapid, and accurate diagnosis is needed to improve development of treatment approaches, enhance prognosis and control dissemination [16].

Direct parasitological diagnosis of *T. gondii* involves the detection of tachyzoites or tissue cysts by direct microscopy or isolation in cell culture. However, pathologic tissue examination or strain isolation are mostly used for diagnosis of animal infection, less frequently for that of human toxoplasmosis. Currently, nucleic acid and specific antibody assays of *T. gondii* are the most commonly used techniques in clinical settings [14]. Particularly, the serum IgM/IgG antibody test has been widely used as a primary screening method for toxoplasmosis infection. However, this method may fail to detect specific anti-*T. gondii* antibodies during the active infection phase in animals, because these antibodies may only be produced after several weeks of parasitemia [10]. In recent years, PCR, real-time PCR, and nested-PCR assays have become essential tools for the molecular diagnosis of *T. gondii*. These PCR-based amplification techniques have revealed good sensitivity and specificity, with real-time PCR with probe hybridization reported to be the most sensitive assay [25]. Nevertheless, widespread clinical application of these techniques has been limited by several factors, including the need for sophisticated instruments and well-trained personnel.

Since its discovery, the loop-mediated isothermal amplification (LAMP) technique has attracted considerable attention because of its simple amplification conditions and high-efficiency amplification. The technique is based on strand displacement activity of *Bst* DNA polymerase with two pairs of specially designed primers, at a constant temperature of around 60–65°C, and a set of amplification products consisting of stem-loop structures containing repetitive target sequence forms [23]. LAMP products can be confirmed by the turbidity of the resulting magnesium pyrophosphate by visual inspection, or addition of a fluorescent dye to the reaction system which makes the product visible under UV light [27]. LAMP has several advantages over traditional PCR, hence it is at the forefront of research in the search for new diagnostic tools for parasitic diseases [10]. During toxoplasmosis diagnosis, a series of LAMP-based assays, targeting B1 gene or 529 bp repeat sequences, internal transcriptional spacer sequences (ITS-1), as well as 18S rDNA sequences, have been established. In addition, detection of LAMP products has further been optimized by combining probe hybridization [1], ELISA [24], and lateral flow dipstick (LFD) [21], which have subsequently improved the sensitivity and specificity of the LAMP assay. Among these optimizations, LAMP-LFD offers optimal detection, and was subsequently applied for detection of parasites and microbes, such as *Mycoplasma ovipneumoniae* [35], *T. gondii* [18], *Babesia bovis* and *Babesia bigemina* [33], canine parvovirus [26], and the African trypanosome [22]. Generally, LAMP-LFD is based on the principle that biotin-primers biotinylate an amplification product while fluorescein isothiocyanate (FITC)-labeled probes de-hybridize, to simultaneously double-label the product, and allow their capture by anti-FITC antibodies on a lateral flow dipstick. The conventional LAMP-LFD method requires opening of reaction tubes to allow addition of the reaction product to the LDF, after completion of LAMP amplification. However, LAMP’s efficient amplification mechanism makes it highly sensitive and prone to aerosol contamination from previous LAMP reactions. These contaminants serve as a template for repeated amplification, leading to inaccurate false-positive results [28, 29]. To address this problem, we designed a simplified, portable, and closed LAMP-LFD format. We targeted the 529-repeated element of *T. gondii*, then developed a new sensitive and simple assay based on LAMP-LFD in a hermetic device for detection. This simple device only requires a regular laboratory water bath and results can be simply read-out. We validated our established LAMP-LFD’s performance by detecting *T. gondii* tachyzoites in genomic DNA extracted from blood samples of stray cats and dogs across Zhejiang province.

## Materials and methods

### Ethics statement

This study was performed in strict adherence to the recommendations in the Guide for the Care and Use of Laboratory Animals, and according to the Animal Ethics Procedures and Guidelines of the Chinese National Institutes of Health. Experimental procedures were approved by the Institutional Animal Care and Use Committee (IACUC) of the Zhejiang Academy of Medical Sciences (approval number: 2018-102).

### Strains and samples

*Toxoplasma gondii* tachyzoites (RH strain), preserved at our laboratory, were used in this study. Briefly, the tachyzoites were aseptically cultured *in vitro*, by serial passages in Vero cells, as previously described [17]. Blood samples were collected from stray dogs and cats, intravenously using a syringe with assistance from experts at the animal protection base of Zhejiang Small Animal Protection Association. A total of 318 blood samples were collected from Zhoushan, Deqing, Lishui, Yiwu, and Wenzhou regions of Zhejiang province. These samples were collected by experienced staff from the animal hospital. All samples were anti-coagulated by EDTA-K2 and DNase inhibitors were added to prevent DNA degradation, and stored at 4°C until nucleic acid extraction within three days.

### DNA extraction

Genomic DNA was extracted from the positive control using an Animal Genomic DNA Quick Extraction Kit (Beyotime, Shanghai, China), according to the manufacturer’s instructions. DNA from anticoagulated blood samples was extracted via magnetic bead adsorption using a KBM Blood Genomic DNA Extraction Kit (KBM, Hangzhou, China). Due to the suspected low amount of circulating DNA in these animals whose status infection was unknown, each sample was divided into three 200μL parts, eluted by 30μL elution buffer in order to improve DNA yield and detection rate. Each replicate was then tested and a positive result for any of the sections indicated that the sample was positive. DNA concentration was determined using a NanoDrop spectrometer (Thermo Fisher Scientific, MA, USA), then stored at −20°C until use.

### Design of primers and probe

**Figure 1:**
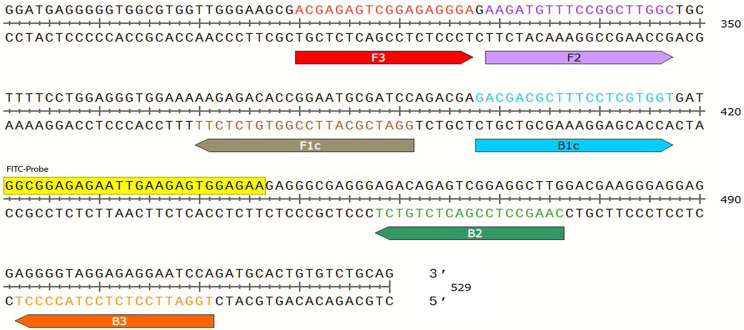
Nucleotide sequence of 529 showing the set of primers and the probe. The sequences marked with red, purple, brown, blue, green, and orange represent primers F3, F2, F1c, B1c, B2, and B3, respectively. The forward inner primer (FIP=F1c−F2) was labeled with biotin at the 5′ end, with amplification in the 5′–3′ direction. The yellow module denotes the sequence of FITC-probe between primer B1c and B2.

Previous studies have reported the B1 gene (GenBank AF179871) and 529-bp repeated element (GenBank AF146527) as the potential optimal targets for *T. gondii*. Particularly, the B1 gene has been extensively used for molecular detection of *T. gondii*, has 35 copies in the *T. gondii* genome [4]. On the other hand, the 529-bp repeated element is a recently discovered target gene, with up to 300 copies, which offers more sensitivity and specificity during detection [12]. Since success of LAMP amplification depends on designing the ideal primers for target the gene, we employed the online LAMP primer designing software Primer Explorer V3 (http://primerexplorer.jp/e) and designed a set of specific oligonucleotide primers targeting the 529-bp repeated element of *T. gondii*. We labeled the 5′ end of the forward inner primer FIP with biotin, and labeled the probe with fluorescein isothiocyanate (FITC). This was designed between primers B1c and B2 for molecular hybridization detecting FITC-biotinylated LAMP product (Fig. 1). The designed primer sequences are listed in Table 1.

**Table 1:** Primer sequences used for PCR and LAMP.

Primer	Sequences (5′→3′)	Amplicon sizes (bp)
LAMP		
F3	ACGAGAGTCGGAGAGGGA	202
B3	TGGATTCCTCTCCTACCCCT	
FIP (F1c−F2)	GGATCGCATTCCGGTGTCTCTTAAGATGTTTCCGGCTTGGC	
BIP (B1c−B2)	GACGACGCTTTCCTCGTGGTCAAGCCTCCGACTCTGTCT	
FITC-Probe	FITC-GGCGGAGAGAATTGAAGAGTGGAGAA	
PCR		
F	ACGAGAGTCGGAGAGGGA	202
R	TGGATTCCTCTCCTACCCCT	

### The LAMP reaction system

LAMP was performed according to a previously reported method [17, 23, 28]. Our optimized LAMP reactions were performed in total volumes of 25μL, comprising 2–4μL of genomic DNA, 12.5μL of 2X reaction buffer (1.6 M betaine, 40mM Tris-HCl (pH 8.8), 20mM KCl, 20mM (NH4)_2_SO_4_, and 0.2% Tween 20), 5 pmol of each of the F3 and B3 primers, 40pmol each of the BIP and biotin-FIP primers, 1μL of Bst 2.0 WarmStart^®^ DNA polymerase (New England Biolabs, Beijing, China), 8.4mM MgSO_4_ (New England Biolabs, Beijing, China), and 1.2μM dNTPs (New England Biolabs, Beijing, China). Amplifications were performed in a water bath, maintained at a constant temperature of 65°C for 1 h, and the resulting LAMP products checked on a 1.5% agarose gel. In a comparative study, 1μL SYTO13 (ThermoFisher, Beijing, China) was used as the fluorescent dye for real-time LAMP, with amplification performed on a CFX96 Touch™ Real-Time PCR Detection System (Bio-Rad, Hercules, CA, USA). The amplification products were determined by the acquisition of the fluorescent signal.

### LAMP-lateral-flow-dipstick (LAMP-LFD)

**Figure 2:**
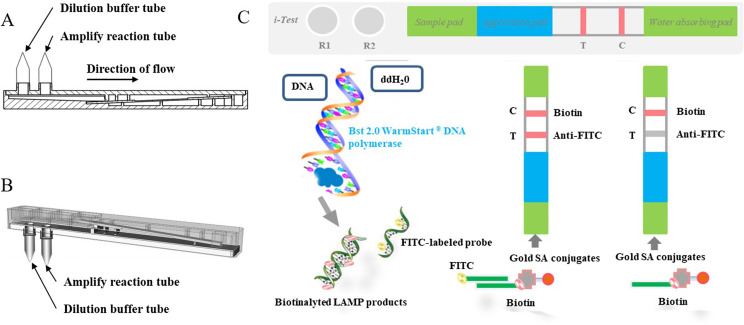
The LAMP-LFD device and principle. (A) Side view of LAMP-LFD equipment. (B) Schematic representation of the LAMP-LFD model. (C) Schematic representation of the working principle of LAMP-LFD. *Cryptosporidium parvum*, *Plasmodium vivax*, *Leishmania donovani*, *Entamoeba histolytica*, *Trypanosoma evansi.*

To detect LAMP products, we designed a universal rapid detection equipment, known as LAMP-Lateral-Flow-Dipstick (LAMP-LFD), by combining the LAMP reaction and lateral flow dipstick (Fig. 2A). The integrated equipment has a micro-amplify reaction tube, for LAMP reaction, and another tube containing nucleic acid dilution buffer with the lateral-flow-dipstick module for LAMP product capture. The LFD detection module comprises a plastic grooved pedestal and a hermetic plastic cover that contains a visualization window and two connectors. The lateral-flow-dipstick, which is set on the plastic grooved pedestal, is composed of sample and application pads, test and control lines, and a water-absorbing pad. In addition, the application pad, as well as test and control lines, are covered with gold-streptavidin (SA) conjugates, an immobilized anti-FITC mouse monoclonal antibody and biotin, respectively. Before starting the assay, the user only needs to connect the micro-amplify reaction tube with the reserved connector after adding the sample to be detected to the LAMP reaction mixture (Fig. 2B). The LAMP-LFD equipment is then incubated in a water bath, maintained at a constant temperature of 65°C for 1h to complete the LAMP reaction. After completion of the desired product amplification, the equipment is turned off, and the FITC-biotinylated nucleic acid products mixed with the nucleic acid dilution buffer. The mixture flows towards the lateral-flow-dipstick, combines with Gold-streptavidin (SA) conjugates to form a triple-labeled complex when it flows through the application band, then moves up the strip. Consequently, it is captured by the immobilized anti-FITC antibody (test line). The biotinylated FIP primer binds to the Gold-SA conjugates to form a double complex without FITC and is trapped at the immobilized biotin (control line) (Fig. 2C). Read-out A positive result is evidenced by test and control lines, which are both visible through the read-window on the cover. Conversely, only the control line is visible in case of a negative result.

### Determination of LAMP-LFD specificity

The aforementioned LAMP and LAMP-LFD protocols, as well as their procedures, were executed to verify specificity of LAMP. Briefly, the template was replaced by genomic DNA extracted from *Cryptosporidium parvum*, *Plasmodium vivax*, *Leishmania donovani*, *Entamoeba histolytica*, and *Trypanosoma evansi*, and the LAMP amplicons confirmed via gel electrophoresis.

### Evaluation of LAMP-LFD sensitivity

LAMP-LFD sensitivity was evaluated against 10-fold serial dilutions of a positive control template (genomic DNA of *T. gondii*). These dilutions ranged from 1 ng to 0.01 fg, with nuclease-free water included as a negative control. To validate our results, each concentration was tested three times. We also performed a polymerase chain reaction (PCR) as described before, using the outer forward (F3) and reverse (B3) primers as upstream and downstream primers, respectively. PCR reactions were performed in 25 μL reaction volumes, comprising 12.5 μL 2X Master Mix (Tsingke, Beijing, China), 5 pmol of each forward and reverse primers, and 2 μL of the template. Amplification was performed as follows; initial denaturation at 94°C for 5min, followed by 30 cycles of denaturation at 94°C for 45s, primer annealing at 53°C for 30s, extension at 72°C for 30s, and a final extension step at 72°C for 10min. PCR products were visualized on a 1.5% agarose gel, stained with Gel-Red (Beyotime, Beijing, China).

### Application of the developed LAMP-LFD device for detection of *T. gondii*

We tested the established LAMP-LFD device for detection of *T. gondii* in blood samples collected from stray animals. Briefly, we extracted genomic DNA from 318 blood samples collected from dogs and cats across Zhejiang Province, China, then used LAMP-LFD and PCR to amplify the 529 gene for detection of *T. gondii*.

## Results

### Specificity of the established LAMP-LFD method

**Figure 3:**
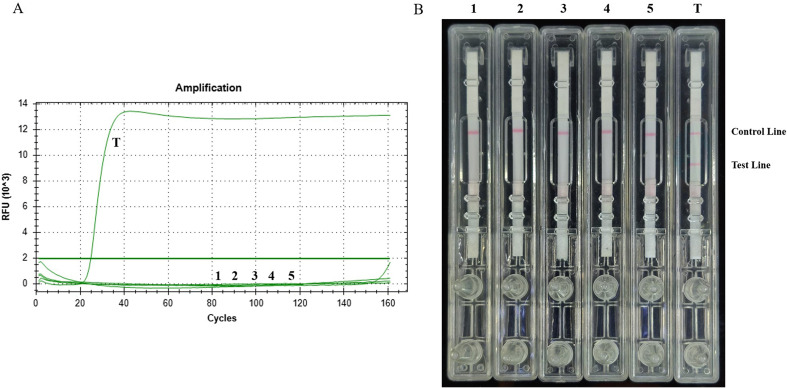
Specificity of LAMP and LAMP-LFD detection methods. (A) Curves for real-time LAMP. (B) Visual inspection of LAMP-LFD. (1) *C. parvum*; (2) *P. vivax*; (3) *L. donovani*;(4) *E. histolytica*; (5) *T. evansi*; (6) *T. gondii* (RH).

We tested specificity of LAMP and LAMP-LFD methods using genomic DNA samples from *Cryptosporidium parvum*, *Plasmodium vivax*, *Leishmania donovani*, *Entamoeba histolytica*, *Trypanosoma evansi*, and *T. gondii* (RH). Only *T. gondii* showed amplification, using both methods, while the other DNA templates showed no signals (Figs. 3A and 3B), indicating that the established method has good specificity.

### Sensitivity of LAMP-LFD and PCR

**Figure 4:**
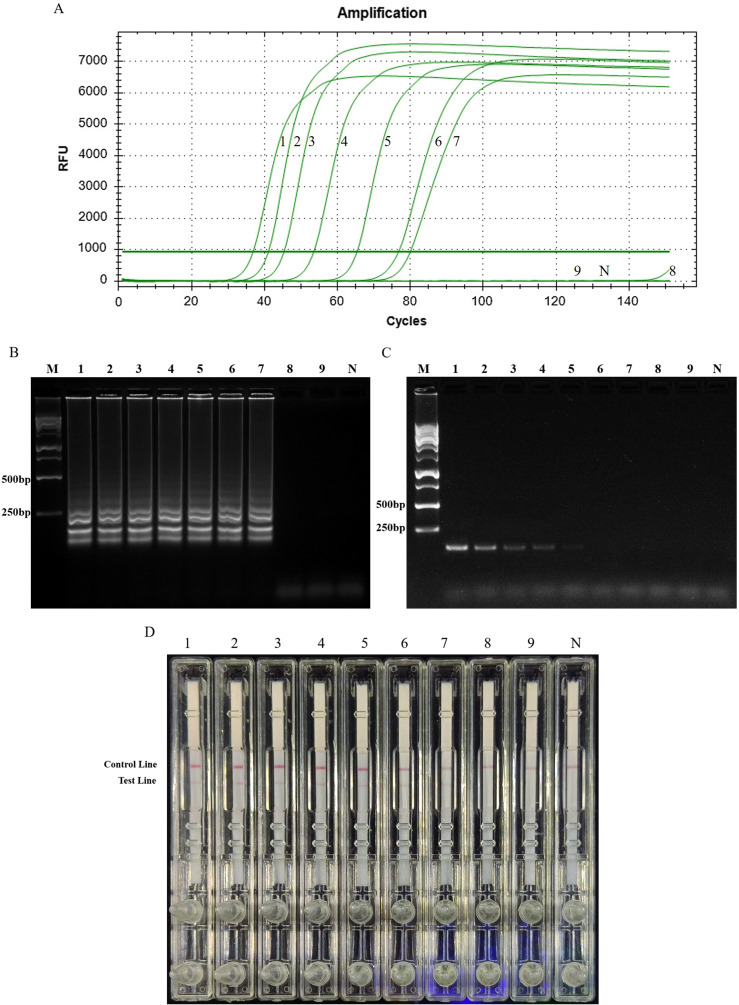
Comparative results of sensitivity for LAMP-LFD and PCR. (A) Signal curve of real-time LAMP. (B) Agarose gel electrophoresis of *T. gondii* LAMP products. (C) Agarose gel electrophoresis of the *T. gondii* PCR products. (D) Visual inspection of LAMP-LFD. Lane M, DNA ladder marker; Numbers 1–8 represent 1 ng, 100 pg, 10 pg, 1 pg, 100 fg, 10 fg, 1 fg, 0.1 fg, 0.01 fg of *T. gondii* DNA, respectively; lane N, negative control.

Next, we performed the sensitivity analysis of LAMP-LFD using different concentrations of genomic DNA extracted from *T. gondii*. Template with a 10-fold concentration, diluted from 1 ng to 0.01 fg, was subjected to real-time LAMP and LAMP-LFD. Signal curves from real-time LAMP showed that the detection limit at 1h was 1 fg genomic DNA of *T. gondii* (Fig. 4A). A similar result was obtained in LAMP-LFD (Fig. 4D). Conventional PCR results revealed a minimum detectable concentration of 100 fg (Fig. 4C).

### Detection of *T. gondii* in stray dogs and cats

**Figure 5:**
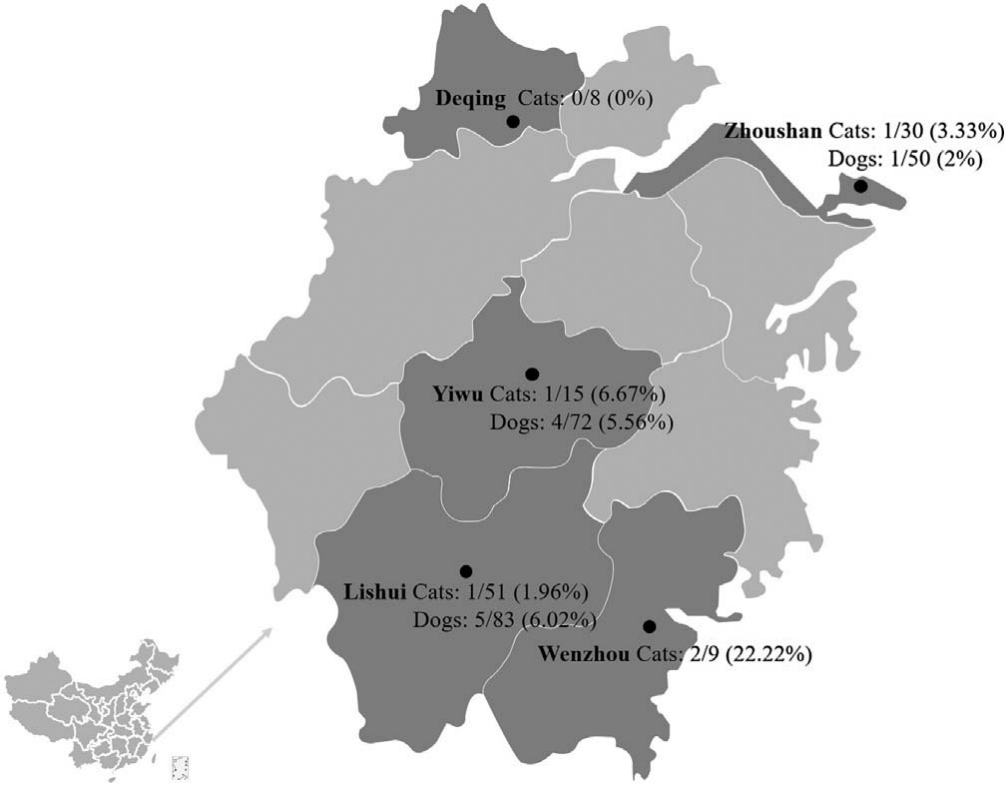
Rates of *T. gondii* LAMP-LFD positive in the blood of stray cats and dogs in five cities.

The LAMP-LFD method detected *T. gondii* DNA in 4.72% (15/318) of the blood samples of stray cats and dogs, and the positive rates of *T. gondii* in the blood of stray cats and dogs in various locations are shown in Table 2 and Figure 5. Higher positive rates were detected by LAMP-LFD (4.72%, 15/318) than by PCR (0.63%, 2/318), as shown in Table 3.

**Table 2:** Stray cat and dog samples detected by LAMP-LFD.

City	Cats		Dogs	
	Positive samples	Total	Positive samples	Total
Deqing	0	0	0 (0%)	8
Zhoushan	1 (3.33%)	30	1 (2%)	50
Lishui	1 (1.96%)	51	5 (6.02%)	83
Yiwu	1 (6.67%)	15	4 (5.56%)	72
Wenzhou	2 (22.22%)	9	0	0
Total	5 (4.76%)	105	10 (4.69%)	213

**Table 3:** Comparative results of conventional PCR and LAMP-LFD assay for the detection of *T. gondii* in the blood of stray cats and dogs in Zhejiang province.

Test methods	Blood samples (*n*=318)	
	Positive	Negative
Conventional PCR	2 (0.63%)	316 (99.37%)
LAMP-LFD	15 (4.72%)	303 (95.28%)

## Discussion

Developing early, rapid, and cost-effective diagnostic methods suitable for economically deprived areas and field testing is essential for early screening, prevention, control, and treatment of toxoplasmosis, owing to its global burden, serious consequences, and lack of effective anti-toxoplasmosis drugs. LAMP is a molecular amplification technique characterized by high sensitivity and specificity, capable of amplifying several copies of nucleic acids to 10^9^ times. Consequently, it has gradually become an alternative to PCR methods, for molecular diagnosis of multiple pathogens. LAMP mainly comprises the *Bst* DNA polymerase with strand displacement activity and a set of four primers that recognize six distinct sequences of the target fragment [23]. LAMP has been widely applied in the diagnosis of toxoplasmosis. With regards to target genes, Burg et al. [4] first proposed use of the B1 gene, that has 35 copies, as a target for molecular diagnosis of toxoplasmosis. Thereafter, the 529-bp fragment became a preferred target owing to its large copy number (up to 300 copies) [12]. Based on these, we designed a set of optimal primers targeting the 529 element for detection of *T. gondii*. Our previous work optimized the LAMP system and reported the optimal reaction temperature [17]. Generally, LAMP products can be confirmed by gel electrophoresis, turbidity measurement of magnesium pyrophosphate, and fluorescent dye method, among others. Since some methods, such as electrophoresis are tedious, previous studies have developed and applied a faster and simpler method for chromatographic lateral flow dipstick (LFD) format to reveal LAMP products [15, 33, 34]. Particularly, LAMP-LFD is more specific for products obtained from molecular probe hybridization techniques, as well as biotin and fluorescein labeling of LAMP products in combination with double-sandwiching.

In the present study, we applied the LAMP and LFD method for product detection. Specifically, we used biotin-labeled internal primers and FITC-labeled probes to generate the ends of the stem-loop structure of respective biotin- and FITC-labeled LAMP product. Thereafter, we captured and visualized the product as it flowed through the anti-FITC antibody region of the lateral flow dipstick. This is more specific and sensitive than both magnesium pyrophosphate turbidity measurement and fluorescent dye methods. Our results indicated that this LAMP-LFD method can detect a template of genomic DNA down to 1 fg in 1h, hence detecting *T. gondii* RH strain, and has no cross-reaction with *Cryptosporidium parvum*, *Plasmodium vivax*, *Leishmania donovani*, *Entamoeba histolytica*, and *Trypanosoma evansi*. Previous studies have reported comparable results using LAMP and LAMP-LFD. For example, Lin et al. reported a detection limit of 10 fg on target 529 element using LAMP [20], whereas Lalle et al. found that LAMP-LFD could detect *T. gondii* oocysts down to 25 oocysts/50g in ready-to-eat baby lettuce [18]. In addition, Fallahi et al. reported a detection limit of 1 fg in *T. gondii* DNA via 529-LAMP [8]. Taken together, these findings indicate that our LDF method guarantees equal detection potency but with better specificity because only biotin- and FITC- amplicons resulting in a band detectable by the LFD strips. In addition to the LAMP method, many other molecular diagnostic methods have been reported for the detection of *T. gondii*, although showing different detection sensitivities. Our results suggest that the LAMP method exhibits higher sensitivity than conventional PCR where the latter may give rise to false negative results. In 2017, Wu et al. used the recombinase polymerase amplification combined with lateral flow strip (RPA-LF) method for the detection of *T. gondii* in the environment and could achieve 0.1 oocysts per reaction tube; nested PCR had a detection limit of 1 oocyst/reaction (*T. gondii* genome size was calculated considering a haploid genome size of 70 fg, and there were 8 haploid genomes in each sporulated oocyst) [31]. In an experiment comparing the performance of LAMP with qPCR for *T. gondii*, Durand et al. reported that the qPCR method could detect 10–100 oocysts/2g of mussel tissue sample [7]. The limit of detection of this qPCR was reported to be 10 fg of *T. gondii* tachyzoite gDNA in Lalle’s study [18]. These results need to consider the effect of DNA extraction efficiency on the true amount of template that was put into the reaction, in addition to the limitations of the analysis of the assay itself. However, this does not prevent us from concluding that the LAMP-LFD method is superior to the reported qPCR and PCR methods in terms of detection efficiency.

The molecular detection of *T. gondii* is not only limited by the detection method, but is also related to the stage of infection of the animals to be tested. Hegazy et al. compared detection rates of *T. gondii* in blood samples of mice at different stages after infection with an ME49 strain [10]. According to results, LAMP revealed 18 positives, out of the 20 examined samples, on the seventh day post-infection. However, both PCR and LAMP failed to detect *T. gondii* in blood samples 56 days post-infection. In addition, both LAMP and routine PCR, targeting the 529 bp RE gene, did not detect *T. gondii* in DNA extracted from blood of mice during the chronic phase of the disease, which can be attributed to progressive decrease in parasitemia levels as infection continues [10]. Taken together, these findings indicate that LAMP-based detection of toxoplasma is limited to the early stages of infection and is difficult to detect across peripheral blood at the quiescent bradyzoite stage. This explains our low detection rate of *T. gondii* in blood samples of stray dogs and cats across Zhejiang province. It is possible that the sampled stray animals may have been already at an advanced stage of infection, rather than the parasitemia stage.

Contamination by aerosol residues is an unavoidable obstacle in the field application for molecular diagnosis, although this has received limited research attention. LAMP-based amplification generates a large number of amplicons over a short period of time, thus greatly increasing the risk of aerosol contamination. Similarly, the LAMP-LFD technique typically requires opening the LAMP reaction tube for subsequent LFD testing, which subsequently introduces the risk of aerosol contamination and leads to false-positives. To minimize the risk of contamination, previous studies have proposed the use of sterile pipetting and LAMP partitioning during reactions [17]. Xu et al. and Hong et al. attempted to load a DNA fluorescent dye into a tin foil, microcrystalline wax-dye capsule then preloaded it into a LAMP reaction tube, which was centrifuged to mix the dye with the product and develop the color after completion of the LAMP reaction [13, 32]. However, how to avoid contamination during LFD testing remains unknown. In the present study, we report a first integral hermetic LAMP-LFD device application for *T. gondii* detection. The operator only needs to prepare the LAMP reaction system in PCR tube, according to standard procedures, connect the reaction tube to the device interface, then complete the LAMP amplification in a water bath at 65°C. The LFD assay is performed by turning the device upside down, to mix the LAMP amplification product with the diluent, then laying the device flat to allow the mixture to flow onto the strip. The results can be visually read-out in the viewing window after 5min. The whole testing process is carried out in the device, which ensures an air-tight environment and minimizes the chances of aerosol contamination. The only equipment required is a water bath, which makes this LAMP reaction a simple, cost-effective and suitable method for use in minimally equipped laboratories as well as field settings. Therefore, the LAMP-LFD device designed in this study is universal. First, this LAMP-LFD assay can detect *T. gondii* in cat or dog specimens, but may be extended to humans and even more broadly to warm-blooded animals, food samples, etc. Likewise, the detection process for other pathogenic microorganisms is much easier by simply replacing the primers specific for other targets for the LAMP method. Second, this device is generalizable to isothermal amplification of nucleic acids, including the latest RPA isothermal amplification technology, which allows for rapid identification of assay products while avoiding diffusion contamination of amplification products.

The optimized LAMP-LFD method positively detected *T. gondii* DNA in blood samples of stray cats and dogs. In 2012, Wang et al. investigated anti-*T. gondii* antibodies, circulating antigens, and DNA in 145 stray cats in Shanghai, and the results showed that the seropositivity rate of *T. gondii* in the stray cat population was 11.7%, the circulating antigen positivity rate was 5.5%, and the circulating DNA positivity rate was 5.71% [30]. This result was also close to our LAMP-LFD assay results. As the final host of *T. gondii*, cats can excrete infective oocysts, which can contaminate the environment, food and water and are sources of contamination for *T. gondii* transmission. Therefore, the survey of *T. gondii* infection rates in stray cats is essential for prevention and control in public health and the interruption of *T. gondii* transmission. In 2016, a meta-study investigated the seropositivity rate of *T. gondii* in pet dogs in mainland China [9]. The results showed that toxoplasmosis was common in pet dogs in mainland China, with a minimum seropositivity rate of 5.8% and a maximum of 16.8%, which suggests that the owner takes control measures by reducing human–dog contact and thus reducing exposure to *T. gondii*. At present, there are still no breakthroughs in developing effective drugs and vaccines for the prevention and control of *T. gondii* in animals. Therefore, the key to prevention and control is to eliminate the source of infection and cut off the transmission route of the disease. As the economy grows and the standard of living improves, people may begin to adopt stray animals from animal protection facilities as pets. The status of *T. gondii* infection in these animals is unknown, which may put pet owners at increased risk for infection. The results of the study provide data for the prevention and control of toxoplasmosis from stray cats and dogs to humans.

In conclusion, we developed a novel *T. gondii* detection assay, based on a closed-device design test format. The LFD assay can be performed directly, following completion of LAMP reactions. Our results indicate that the developed LAMP-LFD device is a reliable and portable diagnostic tool for detecting *T. gondii*. Particularly, the device has good airtightness, exhibits excellent sensitivity and specificity in sample testing, and is therefore suitable for under-equipped laboratories and primary health care facilities. It is expected to enhance clinical diagnosis and epidemiological investigations targeting *T. gondii.*
